# Looking under the hood of executive function impairments in psychopathology: A commentary on “Advancing understanding of executive function impairments and psychopathology: bridging the gap between clinical and cognitive approaches”

**DOI:** 10.3389/fpsyg.2015.01170

**Published:** 2015-08-06

**Authors:** Alexandre Heeren, Joël Billieux, Pierre Philippot, Pierre Maurage

**Affiliations:** ^1^Laboratory for Experimental Psychopathology, Psychological Science Research Institute, Université catholique de LouvainLouvain-la-Neuve, Belgium; ^2^Division of Clinical Neuroscience, Institute of Neuroscience, Université catholique de LouvainLouvain-la-Neuve, Belgium

**Keywords:** executive function, self-regulation, psychopathology, transdiagnostic processes, attentional processes, top-down attention control

Of critical importance for clinical psychological science is why do people differ in their very ability to govern and regulate their thoughts and behaviors? This concern speaks to the paramount nature of executive function (EF) that encompasses a set of general-purpose cognitive control mechanisms regulating lower-level processes and, in turn, enabling self-regulation and self-directed behavior toward a goal (Banisch, 2009). Consequently, EF has been shown to have far-reaching implications for nearly all of our daily activities. At a theoretical level, EF is best characterized as consisting of separable but related processes, with both unique and shared individual differences (Miyake and Friedman, 2012). This unity/diversity model focuses on three aspects of EF, namely updating working memory, shifting, and inhibition, as well as on a common unitary EF ability which spans these components and is posited to be the ability to actively maintain task goals and to use this information to provide top-down support for task-relevant responses (Miyake and Friedman, 2012).

We are thankful to Snyder et al. (2015) for recently engaging in a much-needed comprehensive narrative review about the EF impairments in psychopathology. In their review, they concluded that most psychopathological conditions are associated with fairly uniform deficits in EF tasks, and advocated that this pattern of findings cements the view that there are broad and transdiagnostic impairments in the unitary component of EF, rather than impairments in a few individual specific aspects of EF.

Theoretically, Snyder et al. (2015) also upheld that their conclusions do conform to others who posited impairments in the functioning of attentional networks in psychopathology (e.g., Pacheco-Unguetti et al., 2011; Orellana et al., 2012; Maurage et al., 2014). According to the attentional networks approach (Posner and Rothbart, 2007; Petersen and Posner, 2012), attentional system can be subdivided into three functionally and anatomically independent networks, namely *alerting* (allowing to achieve and maintain a state of alertness), *orienting* (allowing to select sensory information by engaging, disengaging or shifting attention from one stimulus to another), and *executive* (involving the top-down control of attention to resolve response conflicts). Because the notion of “executive network of attention” is defined as similarly to common EF, Snyder et al. (2015) argued that psychopathology should thus predominantly be associated with impairment in this network, but neither with the alerting nor the orienting ones. Strikingly, based on this rationale, they also adjudicated that EF impairments in psychopathology are unlikely to reflect lower-level attentional difficulties. We argue that these conclusions are unjustified.

First, a strict reading of their account is at odds with previous evidence of alerting and orienting networks impairments in a wide range of distinct psychopathological conditions (e.g., Lundervold et al., 2011; Fan et al., 2012; Keehn et al., 2013; Heeren et al., 2015). Moreover, strong associations between the efficiency of these two networks and several key transdiagnostic processes have been recently portrayed. However, they were mostly devoid of any relation vis-à-vis the executive network. For instance, the efficiency of the orienting network predicts the intensity of ruminative thinking (Pẽcher et al., 2011; Tortella-Feliu et al., 2014), which is an established transdiagnostic process involved in the maintenance of several psychopathological conditions (Mansell et al., 2009). Besides, enhanced alerting network functioning is predictive of a greater risk of suppressing distress-related cognitions (Tortella-Feliu et al., 2014), which is known as a maladaptive emotion-regulation strategy (Magee et al., 2012). Finally, situational anxiety and distressing feelings were associated with both alerting (Pacheco-Unguetti et al., 2010) and orienting networks efficiency (Moriya and Tanno, 2009), but not with the executive one. We are thus encouraging researchers to dig far beyond a mere diagnostic-based group-comparison approach to grasp the very nature of the connections among EF, attentional networks, and psychopathology.

Second, because psychopathology is associated with impairments in the executive network of attention, Snyder et al. (2015) argued that this suggests that EF deficits are not due to lower-level attentional difficulties. We believe this inference is too strong. Indeed, there are several publications evidencing lower-level attentional processes impairments (e.g., perceptual processes) across several distinct psychopathological conditions, including autism (e.g., Behrmann et al., 2006), social anxiety (e.g., Rossignol et al., 2012; Peschard et al., 2013), schizophrenia (e.g., Silverstein et al., 2014), depression (e.g., Desseilles et al., 2009), and addictions (e.g., D'Hondt et al., 2014). Although we agree that uncertainty still abounds regarding the elusive connections between lower- and higher-level processes, one cannot rule out the possibility, as recently suggested (e.g., Noël et al., 2013; Peschard and Philippot, 2015), that these interactions do play key roles in the maintenance of psychopathology.

Altogether, although we agree with Snyder et al. (2015) that an audit of EF impairments in psychopathology is timely, our commentary challenges their claim that psychopathology is typically associated with impaired executive network of attention. It also calls for a reconsideration of the role of attention, including lower-level processes, in the conceptualization of EF impairments in psychopathology. Yet it remains to be seen whether a unified theory of the interactions between attentional networks and both diverse and unitary executive components can be achieved, even beyond the case of psychopathology. On the other hand, at a methodological level, extant procedures often used for assessing the structures of EF, such as exploratory and confirmatory factor analyses, are insufficiently robust to delve into the communalities shared by attentional and executive processes. New methods for conceptualizing psychological phenomena as networks of interacting processes, rather than indicators of a latent common variable, have emerged (e.g., Borsboom and Cramer, 2013). Since these methods have more robust validity vis-à-vis construct simulation models that mimic network dynamic of psychological phenomena (e.g., Schmittmann et al., 2013), reasonable next steps would thus be their application for reliably modeling common jointures of attentional networks, EF, and psychopathology.

## Author contributions

AH had the initial ideas and wrote the first draft of the manuscript. All authors then revised the manuscript critically and contributed to and have approved the final manuscript.

### Conflict of interest statement

The authors declare that the research was conducted in the absence of any commercial or financial relationships that could be construed as a potential conflict of interest.
